# Auditory Noise Facilitates Lower Visual Reaction Times in Humans

**DOI:** 10.3390/biology13080631

**Published:** 2024-08-18

**Authors:** Argelia Pérez-Pacheco, Fernando Yael Rodríguez Morales, Khashayar Misaghian, Jocelyn Faubert, Jesus Eduardo Lugo Arce

**Affiliations:** 1 Directorate of Research, Hospital General de México “Dr. Eduardo Liceaga”, Mexico City 06720, Mexico; argeliapp@ciencias.unam.mx (A.P.-P.); frodriguezm811@alumno.uaemex.mx (F.Y.R.M.); 2 Research and Technological Development Unit (UIDT), Hospital General de México “Dr. Eduardo Liceaga”, Mexico City 06720, Mexico; 3 Faubert Laboratory, Université de Montréal, Montreal, QC H3T 1P1, Canada; k.misaghian@sagesentinel.com; 4 Sage-Sentinel Smart Solutions, Onna, Okinawa 904-0495, Japan; 5 Facultad de Ciencias Físico-Matematicas, Ciudad Universitaria, Puebla 72570, Mexico

**Keywords:** reaction time, noise, stochastic resonance, neurotuner

## Abstract

**Simple Summary:**

Noise can positively and negatively affect the systems it interacts with. When the right amount of noise is added to a weak signal, it can make it easier to detect, a phenomenon known as stochastic resonance. Our research focused on applying noise to improve human reaction times. We observed a significant decrease in the reaction times after placing subjects in the beneficial noise branch close to the optimal point. These findings suggest a novel approach to enhancing human performance in tasks that require faster reaction times, such as sports.

**Abstract:**

Noise is commonly seen as a disturbance but can influence any system it interacts with. This influence may not always be desirable, but sometimes it can improve the system’s performance. For example, stochastic resonance is a phenomenon where adding the right amount of noise to a weak signal makes it easier to detect. This is known as sub-threshold detection. This sub-threshold detection’s natural fingerprint is the fact that the threshold values follow an inverse U-shaped curve as the noise intensity increases. The minimum threshold value is the point of maximum sensitivity and represents the optimal point that divides the dynamics in two. Below that point, we can find the beneficial noise branch, where the noise can facilitate better detection. Above that point, the common detrimental noise concept can be found: adding noise hinders signal detection. The nervous system controls the movements and bodily functions in the human body. By reducing the sensory thresholds, we can improve the balance of these functions. Additionally, researchers have wondered if noise could be applied to different senses or motor mechanisms to enhance our abilities. In this work, noise is used to improve human reaction times. We tested the hypothesis that visual reaction times decrease significantly when the subject’s perception is in the beneficial noise branch and closer to the optimal point than outside of this condition. Auditory noise was introduced in 101 human subjects using an interface capable of searching for the right amount of noise to place the subject in the beneficial noise branch close to the optimal point. When comparing the results, the reaction times decreased when the subjects were at the optimal point compared to when the subjects were outside of such conditions. These results reveal the possibility of using this approach to enhance human performance in tasks requiring faster reaction times, such as sports.

## 1. Introduction

In human cognition, the reaction time measures our ability to respond swiftly to external stimuli. It is crucial in various domains, such as athletics, gaming, and military operations. The study of the reaction time has a long history and has become prevalent in human information-processing research [1,2,3,4,5,6,7,8,9,10,11,12,13,14,15,16,17,18,19,20,21,22,23,24,25]. The reaction time reflects the brain’s processing of sensory information and the resulting action. This process involves neural events, from sensing the stimulus to executing a response. Measuring the reaction time provides insights into neural efficiency and cognitive speed. Various types of reaction times exist, such as the simple reaction time, recognition reaction time, and choice reaction time, and assessing them requires multiple tests and sophisticated timing devices in the lab [12,24,26]. There are different types of stimuli and sensory modalities that can affect how quickly humans react [9,27,28,29,30,31,32]. Research findings have revealed the significant impact of factors, such as fatigue, task complexity, sleep deprivation [24,33,34,35,36,37,38,39,40,41,42,43,44,45,46,47], drugs [48,49,50,51,52,53,54,55,56,57,58,59,60,61], age, attention span, overall health [62,63,64,65,66,67,68,69,70,71,72,73,74,75,76], brain injury, concussions, and minor upper respiratory tract infections [8,77,78,79,80,81], on an individual’s response time. Athletes rely on split-second decisions and fast reflexes to excel in their disciplines. Many examples show how exercise can help achieve speedier reaction times [24,36,82,83,84,85,86]. Nevertheless, evidence shows no or the partial effect of exercise on reaction times [87,88,89,90,91,92,93]. Additionally, the importance of reaction times is noted in various fields, including electronic gaming [4,94,95,96,97,98,99] and military scenarios [100,101,102,103,104,105,106]. Advancements in technology, such as virtual reality (VR) and augmented reality (AR) platforms, neurofeedback techniques, brain-training apps, and transcranial direct current stimulation (tDCS), offer promising ways to enhance reaction times [107,108,109,110,111,112,113].

Eldredge and Gould [114] and Parisi [115] emphasized the significance of random fluctuations in complex systems. A complex system can have multiple equilibrium states, like our memory [116,117,118], standard structural glasses [119,120], quantum systems [121,122,123,124,125], neuronal networks [126], collective decision-making in insects [127], and biological population growth dynamics [128]. Parisi’s original model for the climate has two stable states, and environmental noise can induce transitions from one state to another. This result is the origin of the stochastic resonance model for glaciations. Noise can interact with any system and influence it. For instance, adding the right amount of noise to a weak signal can make it easier to detect, which is referred to as sub-threshold detection. The threshold values follow an inverse U-shaped curve as the noise intensity increases [129,130,131,132,133]. Stochastic resonance has been extensively studied in different physical systems [129,134,135], animal population dynamics [136,137], and animal auditory systems [138] and has been extended to human sensory systems and neuronal networks [131,133,139,140,141,142,143,144]. For instance, noise was used to examine the relationship between EEG, event-related potentials, and information processing through an acoustical choice reaction time task [145]. There was a positive correlation between the magnitude of the noise power after the stimulus and the reaction-time performance. In another study [146], the researchers explored the impact of noise on the three-dimensional perception of autostereograms. The signal-to-noise ratio of depth perception is improved at a moderate noise strength level, indicating stochastic resonance. Additionally, half of the participants experienced reduced reaction times to perceive the stereogram when presented with moderate noise levels. Another study investigated stochastic resonance in the memory retrieval speed for multiplication rules [147]. The results show optimal noise levels minimize the response time, with the effects varying based on the task difficulty. 

The impact of ambient sensory stimulation on arousal levels in college students has been investigated [148]. Participants completed a reaction-time task under different light, noise, and tactile stimulus conditions. The interaction of white noise and illumination levels produced a U-shaped function. More recently, it was found that adding background white pixel noise to a random dot motion stimulus improved healthy adults’ ability to discriminate motion direction without affecting their reaction times [149]. The psychophysical responses followed an inverted U-like function of the input noise. In a series of experiments, researchers explored the impact of background white noise on tactile thresholds and reaction times [150]. The study found that the white noise significantly affected both measures. The lowest threshold was achieved with white noise presented binaurally at 70 dB SPL. 

Alvar et al. [151] found a direct correlation between the sound level and autonomic arousal (through skin conductance level) in young adults. They exposed participants to background noise like ventilation equipment at levels ranging from 35 to 75 dBA SPL. The lowest skin conductance was achieved with background noise within 45 to 55 dBA. A recent review indicates that music therapy reduces stress and anxiety in critically ill patients despite variations in the trial designs, timings, and intervention features [152]. Moreover, using the peripheral temperature as an autonomic arousal measure, the association between white noise levels and autonomic arousal is studied [153]. It is shown that an effective auditory noise can modulate the finger temperature in an inverse U-shaped function. For 67% of the participants, 70 dB SPL was the optimal noise to place the subjects at the maximum point of the temperature response. It was shown that the same auditory noise can facilitate the sensitivity of tactile, visual, and proprioceptive system responses to weak signals [154]. The average optimal noise level that facilitated the sensory responses was 69±7 dB SPL. 

Here, our results with 101 human subjects showed that introducing auditory noise reduced the reaction times, indicating the potential for improving human performance in tasks requiring faster reactions, such as sports.

## 2. Materials and Methods

### 2.1. Participants 

One hundred forty-three subjects participated in this study.

#### 2.1.1. Inclusion Criteria

- Youth: 20–35 years without chronic disease diagnosed and BMI < 28
- All subjects should have normal or corrected to normal vision (6/6 or better) with normal stereo acuity, as measured by the Frisby test (40 s arc or better).
- Normal hearing
- Signature of consent under information

#### 2.1.2. Exclusion Criteria

- Uncorrected severe visual or hearing impairment
- Presence of serious neurological disease or disorder
- Presence of not stabilizing comorbidity

Only 101 of the 143 participants completed the study. Of the eligible older adults, 42 were excluded due to missing data. 

Following the Declaration of Helsinki, the institution’s ethics and research committee approved the research protocol (certificate Dl/17/301/03/074). All the study participants provided written informed consent.

### 2.2. Measures and Tests 

#### 2.2.1. Visual Reaction Task

We use a simple response time (SRT) task [155] based on the Deary–Liewald paradigm [156]. In this paradigm, the subject must respond to a single visual stimulus with one response. 

**Figure 1:**
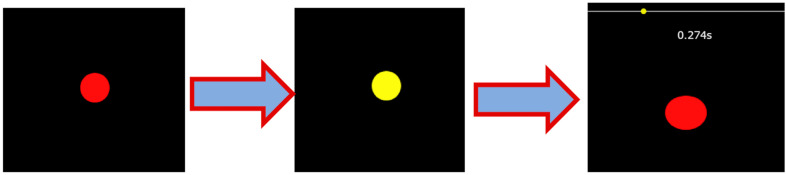
A trial of the visual SRT task. The subject is asked to wait for a red circle with a diameter of 10 mm (**left** panel) to change color to yellow (**central** panel) before pressing the space bar as quickly as possible. Then, the circle changes its color back to red, and the reaction time is displayed on the screen (**right** panel).

Our methodology is straightforward. In the simple reaction time task, the subject is asked to wait for a red circle with a diameter of 10 mm (Figure 1—left panel) to change color to yellow (Figure 1—central panel) before pressing the space bar as quickly as possible. The circle then changes its color to red, and the reaction time is displayed on the screen (Figure 1—right panel).

The task consists of 30 trials (sample data per subject) divided into blocks of five simple response time tasks. Discarding outlier values, which fall outside the typical range, is reasonable as the subject may have been distracted. 

The data collected are saved as a text file for future processing. 

The time between stimuli randomly varies between 1 and 3 s, a crucial aspect of the paradigm. It prevents the task from being too easy because the subject can predict when the stimulus will appear. Figure 1 displays a trail of the visual SRT task.

#### 2.2.2. Crossmodal Stochastic Resonance Interface

The crossmodal stochastic resonance interface used is called NeuroTuner (NTU). NTU (Cognisens Inc., Montreal, QC, Canada) is a computing tool that automatically locates the optimal point (the point of maximum enhancement). The interface introduces auditory white noise to the listeners. 

**Figure 2:**
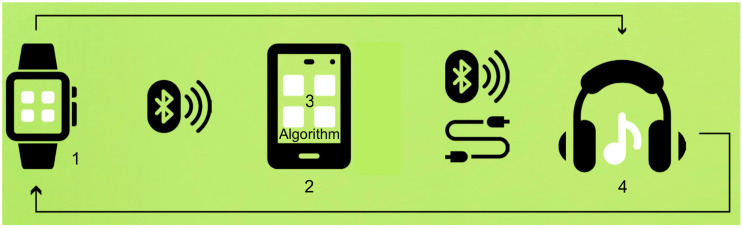
NeuroTuner interface components. A smartwatch is used as the interface to detect HR (1). The HR information is then transmitted to a cellular telephone (2). The cellular phone incorporates an algorithm (3) that modifies the auditory noise levels based on the HRV trend. The auditory input modulates the HRV (4), and this cycle repeats until the optimal point is reached, with the precision of the cellular phone audio card being the limiting factor. The algorithm (3) is described in [153] using HRV instead of temperature.

Figure 2 shows the NTU interface components. The interface consists of a smartwatch that senses HR (1). The HR data are sent to a cellular telephone (2). The cellular phone contains the same algorithm (3) as described in [115], which, depending on the HRV trend, increases or decreases the auditory noise levels that input the subjects (4). The auditory noise modulates the HRV, and the loop continues until the optimal point is located, with a precision limited by the cellular phone audio card. The auditory noise is delivered using a Motorola G fast phone (Motorola Mobility LLC, Chicago, USA). The phone has 15 levels of volume control. By default, the NeuroTuner application sets the maximum volume level at 50% of the phone’s maximum volume level, which in dB SPL (sound pressure level) represents an interval ranging from 40 to 80 dB SPL. The 40 dB SPL represents the sound level measured at the location where the experiments took place. The NTU application’s sound precision limit is 0.5% of the phone’s maximum volume.

Moreover, 40 dB SPL correlates with the sound in a residential neighborhood at night [157], an average home sound level [158], or a quiet environment like a home, office, or library [159]. The disruptions in the testing environment (low-level ambient sounds from outside the testing area) are captured using a large bandwidth microphone. The noise frequency is limited to 2.5 kHz, and they register an intensity of 40 dB SPL. In previous work from our group [154], audiograms between 250 and 8000 Hz were taken in 21 healthy subjects (25–52 years old) with no history of auditory, tactile, visual, motor, or detectable neurological disorders. The hearing in all the subjects was within the normal range, with a group minimum of 8±4.5 dB SPL at 1200±200 Hz. In the present case, the participants’ ages range from 20 to 35. Thus, we should have expected similar hearing thresholds in the present study. However, we performed a quick hearing test to check the participants’ current hearing abilities in the present research. We asked participants if they could perceive the minimum noise level delivered by the algorithm, that is, 0.5% of the phone’s maximum volume plus the external background noise or 40.5 dB SPL. All the subjects could perceive the sound.

Finally, the variation time it takes for the NTU to identify the optimal point in different subjects is 6±2 (one STD) minutes.

**Figure 3:**
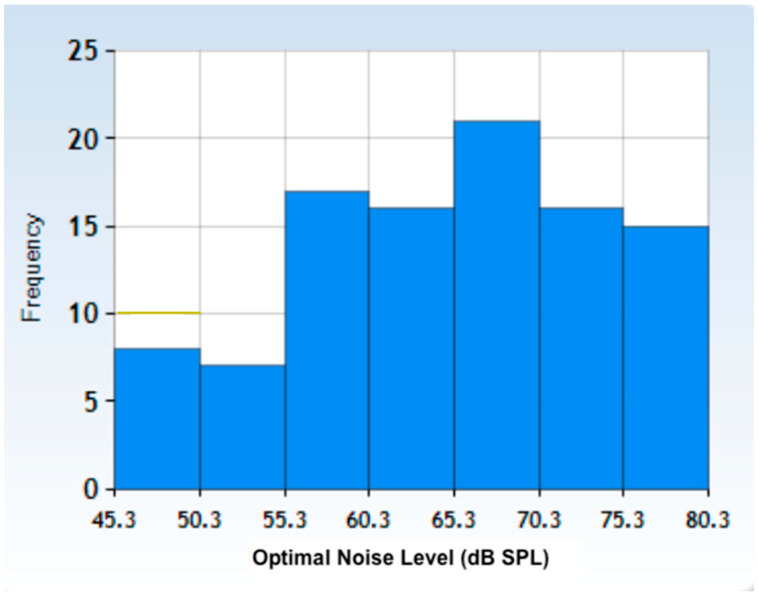
Optimal noise level distribution for the whole population studied here. The distribution has a mean of 65.2 dB SPL, a standard deviation of 9.2 dB SPL, and a median of 66.5 dB SPL. The Kolmogorov–Smirnov normality test statistic D is 0.07, with a *p*-value of 0.71.

Figure 3 displays the distribution of all 101 optimal noise levels. The distribution has a mean of 65.2 dB SPL, a standard deviation of 9.2 dB SPL, and a median of 66.5 dB SPL. The Kolmogorov–Smirnov normality test static D is 0.07, with a *p*-value of 0.71.

#### 2.2.3. Experimental Protocol

All the subjects performed the 30 visual SRT trials (sample data per subject) under two conditions (*NoNTU* and *YesNTU*). The first condition to be used was selected randomly. That is, the order between the *NoNTU* and *YesNTU* trials was randomized, and when the NTU was presented, the reaction time measurement began once the optimal point was located. Thus, there were no added replication benefits. In other words, NeuroTuner took a few minutes (6±2 min) to find the optimal point. Once NTU attained such a condition, the visual SRT task commenced. 

All the subjects sat comfortably in front of a 27-inch monitor at 20 to 28 inches.

### 2.3. Statistical Analysis

The statistical analyses were performed with EXCELversion 11. As is known, reaction time data can be adequately fitted by an exponentially modified Gaussian distribution (or exGaussian) [16,17,160,161], whose probability density function (pdf) is given by:

(1) fx∥μ,σ,τ=1τeμτ+σ22τ2−xτΦx−μ−σ2τσ.

where x is a random variable, *μ* is the Gaussian mean with variance *σ*^2^ and 1/τ is the exponential rate.

#### 2.3.1. Estimate of the Initial Parameters for the exGaussian Distribution

First, the parameters of the distribution can be estimated from the sample data per subject with the method of moments as follows [162,163]:

(2) $$\begin{array}{c} m=\mu +\tau ,\\ s^{2}=\sigma^{2}+\tau^{2},\\ \gamma_{1}=\frac{2\tau^{3}}{{\left(\sigma^{2}+\tau^{2}\right)}^{\frac{3}{2}}}, \end{array}$$

where m is the sample mean, s is the sample standard deviation, and $\gamma_{1}$ is the sample data skewness per subject, respectively. Solving these for the parameters gives:

(3) $$\begin{array}{c} \mu =m-s{\left(\frac{\gamma_{1}}{2}\right)}^{\frac{1}{3}},\\ \sigma^{2}=s^{2}\left[1-{\left(\frac{\gamma_{1}}{2}\right)}^{\frac{2}{3}}\right],\\ \tau =s{\left(\frac{\gamma_{1}}{2}\right)}^{\frac{1}{3}}, \end{array}$$

#### 2.3.2. Optimization of the exGaussian Distribution Parameters

In the second analysis step, we used the set of Equation (3) as the initial values to fit the sample data per subject, utilizing the maximum likelihood estimator (MLE) of a series of parameters $\theta_{i}$ to estimate the distribution parameters. As described in maximum likelihood estimation, for a sample, the likelihood function is defined by [164]:

(4) $$L\left(\theta_{i}\right)=\prod_{j=1}^{N}f(x_{j}∥\theta_{i}),$$

or

(5) $$Log\left(L\left(\theta_{i}\right)\right)=\sum_{j=1}^{N}Log\left(f\left(x_{j}∥\theta_{i}\right)\right)$$

where f is the pdf for the distribution from which the random sample is taken, the exGaussian in the present work. Here, $x_{1},\ldots ,x_{N}$ represent the sample data per subject. The maximum likelihood estimator $\theta_{i}$ is the set of values $\theta_{i}$ that maximizes Equation (5). We can then view the maximum likelihood estimator of the set parameters $\theta_{i}$ as a function of the sample data per subject $x_{1},\ldots ,x_{N}$. In our case, f is given by Equation (1), the set of values $\theta_{i}$ are μ, $\sigma^{2}$, and τ, obtained from Equations (3), and *N* equals 30 the sample data per subject.

We used the generalized reduced gradient method already implemented in EXCEL’s solver to maximize Equation (5). In its most basic form, this solver method looks at the gradient or slope of the objective function as the input values (or decision variables) change. It determines that it has reached an optimum solution when the partial derivatives equal zero. At the end, the process gives the set of values $\mu_{x}$, $\sigma_{x}^{2}$, and $\tau_{x}$, which are the values that optimize the fitting.

#### 2.3.3. Goodness of the Fit

In the third analysis step, we verified the goodness of fit by using Pearson’s chi-square goodness of fit test, which is defined by:

(6) $$\chi_{obs}^{2}=\sum_{j=1}^{N}\frac{{\left(x_{j}-E[x_{j}]\right)}^{2}}{E[x_{j}]},$$

where $x_{j}$ are the observed samples (sample data per subject) and $E[x_{j}]$ their expected values, as obtained using the exGaussian distribution pdf with values $\mu_{x}$, $\sigma_{x}^{2}$, and $\tau_{x}$ estimated in the previous step. For sufficiently large values of N, Pearson’s chi-square test statistic has approximately a chi-square distribution with N−1 degrees of freedom, i.e., $\chi^{2}\left(N-1\right)$ [165]. This test is used to see if a population’s category frequency fits a hypothesized distribution. The null hypothesis is that the exGaussian distribution fits the SRT sample data per subject. This hypothesis is rejected with a 95% confidence if the *p*-value is <0.5. We can reach the same conclusion by looking at the test statistic’s critical value, $\chi_{critical}^{2}\left(0.5,N-1\right)$, whose value should be less than $\chi_{obs}^{2}$ (Equation (6)).

#### 2.3.4. Refinement of the exGaussian Distribution Parameters

The downside of the solution you obtain with the GRG algorithm is that it is highly dependent on the initial parameters and may not be the global optimum solution. The solver will likely stop at the local optimum value nearest to the initial conditions, giving you a solution that may or may not be optimized globally. To refine the optimization process, we used the generalized reduced gradient method again, but we slightly varied $\mu_{x}$, $\sigma_{x}^{2}$, and $\tau_{x}$, keeping the restriction that the *p*-values should be higher than 5%. If the new *p*-value was greater than the previous value, we used the new parameter values $\mu_{x}$, $\sigma_{x}^{2}$, and $\tau_{x}$ for further analysis. On the contrary, we use the previous $\mu_{x}$, $\sigma_{x}^{2}$, and $\tau_{x}$ values.

#### 2.3.5. The exGaussian Mean and Standard Deviation

In the fourth analysis step, we obtained the exGaussian mean $m_{exGaussian}=\mu_{x}+\tau_{x}$ and standard deviation $s_{exGaussian}=\sqrt{\sigma_{x}^{2}+\tau_{x}^{2}}$ for all the subjects under the two experimental conditions, *NoNTU* and *YesNTU*. Then, we created the following distributions $m_{exGaussain}^{NoNTU}$, $m_{exGaussain}^{YesNTU}$, $s_{exGaussian}^{NoNTU}$, and $s_{exGaussian}^{YesNTU}$.

#### 2.3.6. The exGaussian Mean and Standard Deviation Distribution Comparison between the Two Experimental Conditions

Kolmogorov–Smirnov normality tests were used to ensure that the distributions being compared were Gaussians. To compare if there were SRT changes without or with NTU usage, we used *t*-tests. If the distributions did not follow a Gaussian distribution, then Wilcoxon tests were used.

If the *t*-test found an effect of the auditory noise on the visual SRT, we provided the size effect through Cohen’s D.

## 3. Results

Table 1 shows the demographic characteristics of the subjects who participated in this study.

### Visual SRT exGaussian Results

**Figure 4:**
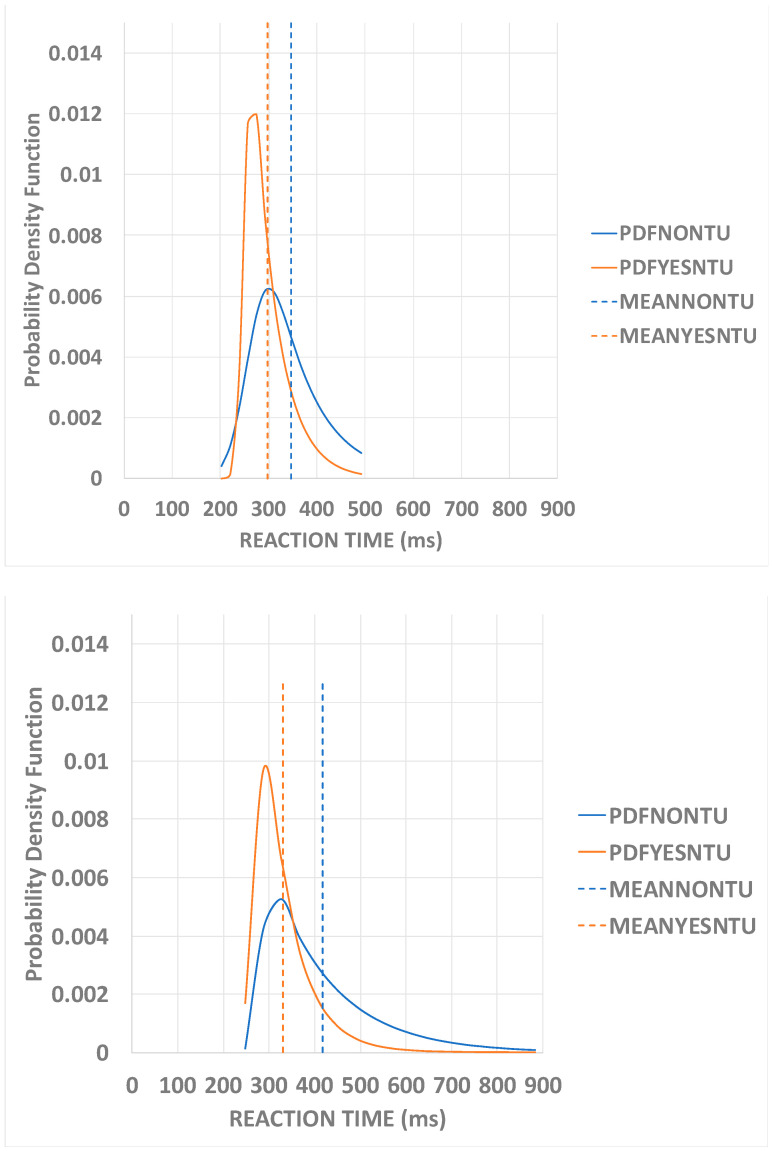
Visual SRT data from two participants show their respective exGaussian distributions when the NTU is used (orange) and not present (blue). The mean of the distribution representing the *YesNTU* condition is shifted to the left of the distribution representing the *NoNTU* for both participants. This result means the auditory noise has influenced the visual SRT, decreasing its value. The broken vertical lines represent the distributions’ means.

Figure 4 displays two participants’ results for the visual SRT task for the two experimental conditions. The use of auditory noise modified the distribution parameters. This change is apparent for both participants. The broken lines locate the means’ distributions. When NTU is used, the mean of the distribution is shifted to the left of the distribution when NTU is not employed. This result means the auditory noise has influenced the visual SRT, decreasing its value. For most subjects, the decrement is true; 83 out of 101 decreased their means, but 18 out of 101 were the opposite.

The table in the Supplementary Materials shows the final mean and standard deviation for the exGaussian distributions for all the subjects and two experimental conditions.

We verified if all the data in the table follow a Gaussian distribution. Kolmogorov–Smirnov normality tests were used for that purpose. $m_{exGaussian}^{NoNTU}$ had a D-value of 0.08787 and p=0.3938, $m_{exGaussian}^{YesNTU}$ had a D-value of 0.11297 and p=0.14056, $s_{exGaussian}^{NoNTU}$ had a D-value of 0.0806 and p=0.50249, and $s_{exGaussian}^{YesNTU}$ had a D-value of 0.13688 and p=0.04114. Consequently, $m_{exGaussain}^{NoNTU}$, $m_{exGaussain}^{YesNTU}$, and $s_{exGaussian}^{NoNTU}$ can be considered Gaussian distributions (*p*-value higher than 0.05), and only $s_{exGaussian}^{YesNTU}$ cannot.

Since $m_{exGaussian}^{NoNTU}$ and $m_{exGaussian}^{YesNTU}$ can be considered Gaussian distributions, a two-tailed *t*-test, with a significance level of 0.05, was performed to compare them and assess whether they were different. The 101 participants who received the NTU intervention ($m_{exGaussain}^{YesNTU}$, *mean* = 0.286, standard deviation = 0.035) compared to the same 101 participants who did not receive the NTU ($m_{exGaussain}^{NoNTU}$, *mean* = 0.314, standard deviation = 0.041) demonstrated significantly lower visual SRT reaction times scores, *t*(200) = 5.31549, *p* < 0.00001. The effect size gives us a standardized way of assessing the *magnitude* of the auditory noise effect over the visual SRT, such as Cohen’s *D*. We obtained a value for *D* equal to 0.8, which can be considered a significant value.

Because $s_{exGaussian}^{YesNTU}$ cannot be considered a Gaussian distribution, we used a two-tailed Wilcoxon test with a significance level of 0.05 to compare $s_{exGaussian}^{NoNTU}$ and $s_{exGaussian}^{YesNTU}$. The 101 participants who received the NTU intervention ($s_{exGaussain}^{YesNTU}$, *median* = 0.051) compared to the same 101 participants who did not receive the NTU ($s_{exGaussain}^{NoNTU}$, *median* = 0.048) demonstrated no significant differences, *W*(100) = 2430, *p* = 0.62414.

**Figure 5:**
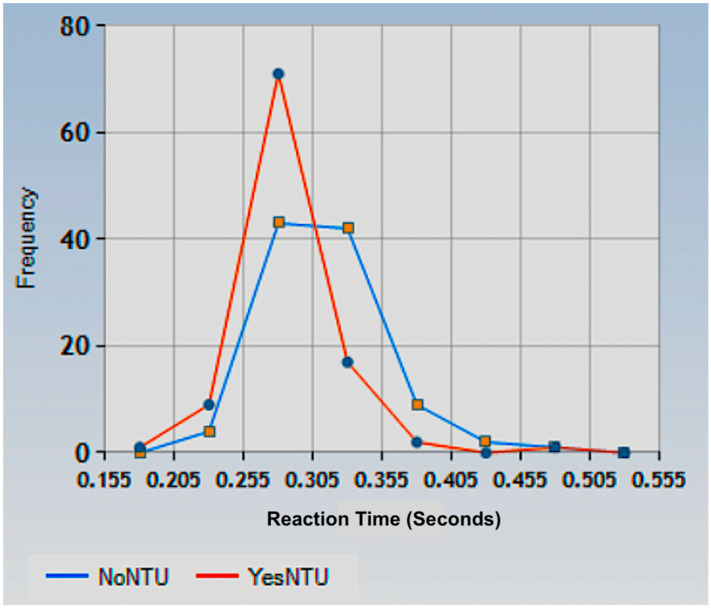
Visual SRT data histograms for two experimental conditions used in this study (NTU present or not) for the population tested here (101 subjects). The whole distribution representing the use of the *YesNTU* condition (*mean* = 0.286, *SEM* = 0.0035) is shifted to the left of the distribution representing the *NoNTU* condition (*mean* = 0.314, *SEM* = 0.0041). This result means the auditory noise has influenced the visual SRT, decreasing its value on average by 28±8 mS (means difference ± one standard error).

Figure 5 shows histograms of the visual SRT data for the two experimental conditions, with and without the NTU, across the 101 subjects. The histogram for the *YesNTU* condition showed a leftward shift in the distribution compared to the *NoNTU* condition. This result suggests that auditory noise has impacted the visual SRT, decreasing on average by 28±8 mS (means difference ± one standard error). The solid lines represent the Gaussian fits.

## 4. Discussion

The recruited participant group was homogeneous regarding the age, education, and gender balance.

We found that the auditory noise influenced the visual SRT, decreasing its value. This result is actual for most subjects; 83 out of 101 (82%) decreased their means, but 18 out of 101 (18%) did the opposite. We showed that auditory noise has impacted the visual SRT in our population by decreasing its average by 28 ± 8 ms, which is higher than the 20 ms obtained with force feedback equipment as described in [109,110]. The statistical significance of the *t*-test tells us that we can be confident that there is an effect. Using a measure of the effect size, like Cohen’s *D*, allows for a standardized assessment of the effect’s magnitude. In the current scenario, the effect size is large, close to 0.8.

What are the mechanisms by which auditory noise decreases the visual SRT in humans?

The most straightforward explanation is that low noise levels can act as a form of “masking noise”, helping to reduce interference from the surrounding environment. On the other hand, higher noise levels can be bothersome and distract the individual, reducing their focus on visual cues. A low noise level can be a helpful background noise, aiding the individual in focusing on the task at hand. Conversely, noise that is too loud can be perceived as annoying. It is important to note that each individual’s hearing threshold is different, so what is considered “low” or “too loud” can vary from person to person. Nonetheless, all the subjects are capable of hearing 40.5 dB SPL, which represents the background external noise level plus the minimum noise level delivered by the algorithm, and the optimal noise level distribution has a mean of 65.2 dB SPL, with a standard deviation of 9.2 dB SPL. It is challenging to believe the masking effect assertion because there is no external noise above 40 dB SPL to mask, but it would be right in the “too loud” part where the noise can be perceived as annoying.

Another possibility to explain this result is using stochastic facilitation [133]. Notwithstanding the assumption that random variations or disturbances reduce performance, they can enhance information processing in non-linear systems. One type of “stochastic facilitation”, called stochastic resonance, has been seen to improve processing in theoretical neural systems and experimental neuroscience. For instance, there is a growing body of empirical literature that suggests that noise at the right intensity may enhance the detection and processing of auditory [166,167,168,169], sensorimotor [150,154,170,171,172,173], and visual stimuli [146,149,154,174,175,176,177], neural synchronization within and between cortical networks [140,145,178], the speed of memory retrieval [147], reaction times, and sensorimotor concurrently [150], autonomic arousal [151,153], proprioception, and physical performance [154,179,180,181,182], central mechanisms of perception [183,184], learning [185], attention performance [186], and audiovisual speech comprehension [187]. Recent research has shown that auditory white noise can significantly enhance memory performance in college students [188]. This enhancement is attributed to the increased attention, alertness, and sensory processing that auditory white noise induces during memorization. These findings suggest that incorporating auditory white noise into learning environments can potentially boost memory performance when remembering and learning visual items. It was also proposed that a universal principle to improve sensory processing is a stochastic resonance facilitated by noise when two senses are involved [189].

Interestingly, using auditory noise can suppress visual perception [190]; it was found that white noise bursts presented through headphones degraded visual orientation discrimination performance. This auditory suppression effect on the visual perception frequently occurred when these inputs were presented spatially and temporally consistently. Past results have shown that the optimal point for the auditory facilitation of visual perception is 75 dB SPL (luminance-modulated stimulus), 70 dB SPL (flicker), 73 dB SPL (luminance-defined stimulus), and 72 dB SPL (contrast-modulated stimulus) [154]. However, the visual suppression described in [190] was found for noise levels of 45 dBA and 65 dBA, and the task is more complex because it involves not only luminance or contrast cues but also spatial orientation or simple visual reaction times, as in this work. This work is essential because facilitation or suppression may depend on task complexity and spatial and temporal congruency between the two sensory modalities.

Additionally, auditory information may facilitate reaction speed in sports [191]. A study with badminton players found that auditory reactions were faster than visual ones but slower than multisensory stimulation. The results suggest that auditory processes can enhance reaction times in sport-specific situations, emphasizing their potential for training interventions in racquet sports. This work emphasizes the importance of the facilitation signal’s audiovisual characteristics context, not only its amplitude and frequency bandwidth.

Moreover, the practical use of this concept is discussed in [139], where the authors found significant evidence supporting the use of stochastic resonance in the development of sensory prosthetics. However, the advancement of stochastic resonance-based technologies varies depending on the sensory modality. The authors recommend further exploration of each modality to maximize the benefits and build upon existing progress. They also highlight stochastic resonance as a potential tool for enhancing current technologies and inspiring innovations.

Nevertheless, one aspect that we must consider if the stochastic resonance is at play here is that random fluctuations in physical systems, at either the microscopic or macroscopic levels, which are referred to as “noise”, are not the same as “acoustic noise” in the auditory system. Therefore, whether “acoustic noise” leads to “neural noise” in the central auditory, visual, and overall nervous systems is uncertain. In our present case, the output of the human system, as measured by the visual SRT, aligns with the stochastic resonance theory. However, U-shaped effects are present in various fields of biology and many aspects of neuroscience.

Furthermore, the current physics-based approach in stochastic resonance research has not fully explored all the potential ways to advance our understanding of the beneficial role of noise in neural systems [133]. A new framework for studying stochastic facilitation in neural systems is needed to better understand the mechanisms already known to neuroscience and explore new areas. This framework could significantly impact other areas of neuroscience and inspire similar efforts to understand other complex systems. Our knowledge of neural systems is not yet fully comprehensive. By integrating theory and experiments to explore the beneficial effects of biologically pertinent noise, we might encourage comparable research endeavors in different aspects of neuroscience. The future is not only full of promise but also compelling and thrilling.

The results discussed above may indicate that stochastic resonance could significantly influence the temporary formation and disintegration of networks of brain areas involved in perception, cognition, and action. The brain’s inherent noise levels change considerably throughout the sleep–wake cycle and its different stages and in reaction to external requirements, controlled mainly by activity in the reticular activating system and the more particular arousal system controlled by the thalamus [192,193]. The formation of neural networks might be influenced by the existing level of neural noise, and stochastic facilitation could be significant in enabling communication within and between brain regions, as the internal noise level affects the synchronization of oscillations that facilitates this communication.

If external noise could synchronize different brain regions to modulate the arousal system, the decrease in the visual SRT would be influenced by the arousal system. Researchers have found that arousal, including muscular tension, is one of the main factors affecting reaction times. The optimal reaction time occurs when the subject has an intermediate level of arousal, whereas the reaction time is hindered when the participant is either too calm or too stressed [24,33,34,36,37,38]. A U-shaped curve represents this relationship, and the point of the minimum reaction time indicates the optimal point. Sports psychologists have traditionally employed the inverted-U hypothesis to explain the arousal-performance relationship, but many researchers have questioned this connection. Presently, there is a shift toward a more comprehensive outlook on the impact of arousal anxiety on performance.

Nevertheless, in one study [194], 104 college students participated in a study where they performed a response time task while riding a bicycle ergometer. The participants were assigned to different arousal groups and were told they were competing for a cash prize. The study found that optimal performance was seen at 60% and 70% of maximum arousal, supporting the inverted-U hypothesis. Somatic anxiety, as measured by the Sport Anxiety Scale, accounted for the significant variance in performance. These findings question the utility of theories that differentiate cognitive and somatic anxiety for predicting performance on simple tasks.

VaezMousavi et al. [45] conducted a study measuring arousal through skin conductance in a continuous performance task. The study revealed that while some participants displayed a traditional pattern of an inverse U-shaped function, others displayed an opposite trend, like 18% of our subjects. In general, an increase in arousal tended to lead to an improvement in reaction time.

Why did all the subjects not decrease their visual SRT when the auditory noise was applied? One plausible explanation is that some individuals may find the auditory noise bothersome. As we are utilizing auditory noise, it is reasonable to suggest that the decibel sound pressure level range we have selected (which is audible) may be perceived as annoying by certain people. The threshold for sound annoyance is multifaceted, and no single predetermined value can conclusively determine it. There have been instances where even soft sounds (e.g., the 35 dBA sound of a toilet flushing from an apartment above) have resulted in high annoyance levels [195]. Annoyance is context-dependent, and under certain circumstances, the white noise level we have employed, which may be tolerable for an average-hearing person, could be considered bothersome. For example, if the noise was perceived to interfere with performance in an experiment where the participant was striving to do well. It is worth noting that participants were subjected to white noise during the experiment. As such, the noise could have been interpreted as an intrusion and irritating at any level for some of them. Interestingly, we found similar results in the past, where in 25% of the studied population, the noise at a fixed auditory level (70 dB SPL) decreased the subjects’ performance. For instance, the tactile thresholds increased when the auditory noise level (70 dB SPL) was present in 25% of the explored population [154].

It is also possible that specific individuals find visual SRT tasks challenging. Studies suggest that stochastic resonance (SR) affects human retrieval speed for arithmetic multiplication rules. An experiment showed an optimal level of acoustic noise that minimizes the average response time. The study also found that the optimal noise level and the magnitude of the SR effect depend on the task’s difficulty [147,179]. Yashima et al. [179] conducted a study on the impact of auditory noise on balance control. They found that auditory white noise, emitted at the detection threshold, significantly increased the standing time of individuals with lower balance (below 10 s) compared to the no-noise trials. However, the noise did not substantially affect individuals with higher balance (above 10 s).

Lastly, Table 1 shows that three subjects had depression, three asthma, two dyslipidemia, and four hypothyroidism. Of these 12 subjects, only three presented the opposite reaction time shifting; that is, they increased their reaction time. In the future, it would be worthwhile to study specific populations, but this is not within the scope of the current article.

## 5. Conclusions

Disregarding the mechanism whereby the SRT decreases, we experimented to examine whether the visual reaction time decreases when the subject’s perception is closer to the optimal point in the beneficial noise branch than outside of this condition. To find the right noise level to place the subject in the beneficial noise branch close to the optimal point, we used an interface known as NeuroTuner to introduce auditory noise to 101 subjects. After analyzing the results, we discovered that the reaction times decreased when the subjects were near the optimal point compared to outside such conditions. These findings suggest that this method could potentially enhance human performance in tasks that require faster reaction times in a more ecological and non-invasive way, such as sports, e-sports, marketing, writing, etc. We aim to test different populations belonging to those activities in future research.

## Figures and Tables

**Table 1:** Demographic characteristics of the population (*N* = 101) that participated in this study.

Variable		
Sex	Female	53
	Male	48
Age (years)	Mean	31
	SD	3
Education	Degree (college)	100%
Degree (college)	Single	96
	Married	4
	Living in common law	1
Hours of Sleep (hr)	Mean	5
	SD	1
Comorbidities	Hypothyroidism	4
	Depression	3
	Asthma	3
	Dyslipidemia	2

## Data Availability

Data are contained within the article.

## Supplementary Materials

The following supporting information can be downloaded at https://www.mdpi.com/article/10.3390/biology13080631/s1.
