# Supplementary material for: Auditory Noise Facilitates Lower Visual Reaction Times in Humans
Source: Biology (Basel). 2024 Aug 18;13(8):631. doi: 10.3390/biology13080631 (PMC11352084; doi:10.3390/biology13080631)
Supplement: Supplementary file 1 [file biology-13-00631-s001.zip › biology-3054453-supplementary.pdf]

Suplementary information

Table S1. exGaussian means and standard deviations for all 101 subjects and two experimental conditions (auditory noise present or not).

$m_{exGaussian}^{NoNTU}$	$m_{exGaussian}^{YesNTU}$	$s_{exGaussian}^{NoNTU}$	$s_{exGaussian}^{YesNTU}$
0.220	0.212	0.018	0.015
0.278	0.275	0.022	0.061
0.264	0.265	0.040	0.032
0.304	0.282	0.067	0.046
0.254	0.261	0.028	0.044
0.379	0.283	0.078	0.045
0.292	0.273	0.051	0.033
0.348	0.256	0.087	0.051
0.292	0.280	0.027	0.033
0.292	0.323	0.027	0.056
0.277	0.261	0.039	0.047
0.457	0.225	0.109	0.074
0.306	0.290	0.030	0.044
0.295	0.251	0.038	0.074
0.293	0.277	0.052	0.043
0.331	0.310	0.063	0.061
0.324	0.285	0.055	0.054
0.293	0.261	0.048	0.039
0.311	0.305	0.031	0.061
0.344	0.303	0.057	0.059
0.239	0.250	0.031	0.034
0.387	0.297	0.097	0.066
0.263	0.272	0.028	0.032
0.452	0.195	0.142	0.071
0.283	0.271	0.036	0.023
0.392	0.304	0.093	0.048
0.273	0.259	0.028	0.034
0.433	0.482	0.107	0.252
0.310	0.290	0.066	0.057
0.313	0.302	0.063	0.072
0.332	0.290	0.058	0.053
0.359	0.336	0.053	0.048
0.311	0.288	0.043	0.042
0.316	0.319	0.043	0.047
0.324	0.287	0.059	0.044
0.330	0.299	0.062	0.045
0.300	0.285	0.043	0.044
0.329	0.325	0.057	0.053
0.284	0.263	0.056	0.053
0.310	0.310	0.049	0.098
0.333	0.299	0.070	0.073

0.267	0.264	0.038	0.046
0.288	0.256	0.051	0.049
0.284	0.276	0.046	0.044
0.292	0.271	0.054	0.065
0.300	0.314	0.025	0.050
0.282	0.269	0.029	0.030
0.278	0.273	0.025	0.030
0.293	0.261	0.052	0.031
0.303	0.279	0.043	0.081
0.309	0.294	0.046	0.069
0.284	0.286	0.036	0.047
0.316	0.299	0.068	0.057
0.338	0.349	0.065	0.082
0.300	0.277	0.041	0.070
0.355	0.367	0.075	0.071
0.352	0.252	0.110	0.047
0.340	0.268	0.070	0.037
0.289	0.242	0.079	0.029
0.291	0.274	0.049	0.038
0.354	0.269	0.077	0.072
0.375	0.258	0.100	0.058
0.337	0.282	0.052	0.026
0.285	0.286	0.043	0.042
0.321	0.298	0.052	0.043
0.317	0.315	0.037	0.061
0.314	0.280	0.051	0.035
0.286	0.267	0.034	0.034
0.307	0.269	0.040	0.035
0.297	0.287	0.031	0.032
0.287	0.328	0.059	0.075
0.403	0.248	0.090	0.037
0.287	0.280	0.032	0.050
0.315	0.272	0.075	0.031
0.302	0.328	0.038	0.041
0.266	0.260	0.030	0.037
0.322	0.281	0.065	0.038
0.348	0.322	0.061	0.089
0.292	0.250	0.034	0.019
0.315	0.276	0.043	0.054
0.319	0.307	0.050	0.065
0.331	0.278	0.069	0.056
0.269	0.273	0.026	0.043
0.334	0.335	0.056	0.106
0.304	0.303	0.043	0.050
0.331	0.301	0.074	0.059
0.326	0.279	0.058	0.066

0.323	0.281	0.061	0.052
0.383	0.363	0.083	0.118
0.328	0.338	0.076	0.062
0.299	0.266	0.044	0.029
0.274	0.268	0.025	0.044
0.356	0.304	0.077	0.058
0.348	0.302	0.057	0.063
0.327	0.314	0.038	0.071
0.274	0.290	0.041	0.049
0.302	0.298	0.045	0.061
0.344	0.266	0.065	0.035
0.291	0.273	0.046	0.044
0.344	0.279	0.048	0.024
0.218	0.212	0.009	0.009

* The exGaussian distribution fit has a p-value higher than 0.05 in all distributions.
